# Enhancement of Cytomegalovirus-Specific Cytokine Production after Modulation of the Costimulation in Kidney Transplant Patients

**DOI:** 10.1155/2019/3926175

**Published:** 2019-02-25

**Authors:** Theresa Dornieden, Benjamin Wilde, Johannes Korth, Kai Werner, Peter A. Horn, Oliver Witzke, Monika Lindemann

**Affiliations:** ^1^Institute for Transfusion Medicine, University Hospital Essen, University of Duisburg-Essen, 45147 Essen, Germany; ^2^Department of Nephrology, University Hospital Essen, University of Duisburg-Essen, 45147 Essen, Germany; ^3^Department of Infectious Diseases, University Hospital Essen, University of Duisburg-Essen, 45147 Essen, Germany

## Abstract

Kidney transplantation is the therapy of choice for patients with end stage renal disease. Due to immunosuppressive treatment, patients are at risk for opportunistic infections. Cytomegalovirus (CMV) reactivation is highly relevant in kidney transplant recipients because it occurs—depending on the serological constellation of the donor and recipient—in more than half of the patients and influences patient outcome. Patients with CMV reactivation show decreased allograft and overall survival. Previous studies could demonstrate that transplant patients often show weak CMV-specific immunity. Besides immunosuppressive treatment, additional mechanisms may reduce CMV-specific immunocompetence such as enhanced negative costimulation. Hence, the aim of this study was to investigate if the function of CMV-specific cells of kidney transplant recipients could be restored by a modulation of costimulatory molecules. To address this question, lymphocytes of kidney transplant patients were stimulated with CMV-specific antigens and incubated with programmed death-ligand 1 (PD-L1), programmed cell death protein 1 (PD-1), or B- and T-lymphocyte attenuator (BTLA) antibodies. Afterwards, the IFN-*γ*, IL-21, and IL-17A production was measured by the ELISpot assay. It could be shown that a blockade of the ligand PD-L1 resulted in an increased CMV-specific IFN-*γ*, IL-21, and IL-17A secretion. The blockade of the receptor PD-1 distinctly enhanced the production of IL-21. BTLA antibodies, however, led only to a marginal increase of CMV-specific IFN-*γ* and of IL-21 production. Experiments in healthy controls could confirm the results of the kidney transplant recipients. Furthermore, they could demonstrate that treatment with the immunosuppressive drug tacrolimus resulted in decreased CMV-specific IFN-*γ* and of IL-21 production. Thus, our study could show for the first time that the blockade of the PD-L1/PD-1 pathway also modulates CMV-specific Th21 and Th17 cell function in kidney transplant recipients. Further studies are mandatory to clarify the role of Th21 and Th17 cells in CMV control of these patients.

## 1. Introduction

Patients with end stage renal disease (ESRD) are dependent on renal replacement therapy. Currently, renal transplantation (RTX) is the first choice for ESRD patients. RTX patients show a survival benefit and decreased morbidity in comparison to age- and sex-matched patients on dialysis as therapy for ESRD. However, RTX patients need to be treated with immunosuppressive therapy following transplantation to avoid allograft rejection. The immunosuppressive therapy leads to an increased risk for opportunistic infections. One of the most common infections is caused by cytomegalovirus (CMV) which may induce fever, leukopenia, interstitial pneumonia or hepatitis [1, 2], or may trigger alloreactivity [1–3]. RTX patients with primary CMV infection or reactivation of CMV show decreased allograft and overall survival [4]. CMV belongs to the family *Herpesviridae* and to the subfamily *Betaherpesvirinae*. In healthy individuals, primary infection with the virus normally proceeds without symptoms [5]. After this primary infection, the virus establishes a lifelong latency in the host [6]. During this latency phase, the virus can reactivate under several circumstances potentially compromising anti-infectious immunity like stress, infection, or inflammation [6, 7]. Different strategies have been developed to reduce the risk for CMV infection/reactivation in RTX patients. Stratification by donor/recipient anti-CMV serostatus is a key to further decide for an appropriate treatment strategy. Despite preemptive or prophylactic antiviral strategies, severe CMV disease occurs in RTX patients [8].

A study by Sester et al. [9] showed that CMV-specific T-cells displayed higher expression of PD-1, resulting in impaired CMV-specific T-cell responses. An inhibition of PD-L1 and PD-L2 could lead to an increased proliferation of these cells [9]. The PD-1 signaling pathway is an inhibitory pathway, which serves for the regulation of early T-cell activation and plays a role in inflammation and autoimmunity [10, 11]. The signaling pathway leads to an inhibited proliferation and a reduced effector function and cytokine production of T-cells [10]. BTLA is another inhibitory receptor, which plays a role in T-cell and B-cell activity [12, 13]. The signaling pathways result in inhibition of T-cell proliferation and cytokine secretion as well as reduced antibody production [13–15]. Blockade of inhibitory pathways has been demonstrated to recover CMV-specific T-cell-derived IFN-*γ* production [16]. Two further cytokines regulating T-cell responses, IL-21 and IL-17A, may also be involved in CMV-specific cellular immunity. IL-21 is a cytokine produced by T-cells and NKT-cells, with the primary role of regulating the function and differentiation of T-cells [17]. It could be shown that chronic CMV infection in aged patients leads to an increased IL-21 secretion. Through this chronic viral infection, the differentiation of naïve CD4^+^ T-cells towards follicular helper T (Tfh) cells is increased and thereby the production of IL-21 as well [18]. The cytokine IL-17A is especially secreted by activated T-cells and plays a role in proinflammatory immune responses [19]. Previous studies could demonstrate an increase of IL-17 production in CMV-positive liver transplant patients in comparison to CMV-negative patients, which shows that the proinflammatory cytokine is involved in CMV infection [20]. Until now, it has not been investigated if Th21 and Th17 cell function can be recovered by the blockade of inhibitory pathways.

In the current study, it was investigated if a blockade of the PD-1 pathway restores CMV-specific production of T-cell-derived cytokines such as IFN-*γ*, IL-21, and IL-17A. In addition, it was studied if a blockade of the recently described inhibitory BTLA pathway may enhance CMV-specific effector responses in RTX patients. Finally, the effect of immunosuppressive drugs combined with an inhibition of the PD-1 pathway was studied in healthy controls. Antibodies against PD-L1, PD-1, and BTLA were titrated in kidney transplant recipients and healthy controls with positive CMV IgG serostatus. To assess T-cell immunity against CMV immediate early antigen-1 (IE-1) and phosphoprotein 65 (pp65), IFN-*γ*, IL-21, and IL-17A ELISpot assays were performed.

## 2. Materials and Methods

### 2.1. Isolation of PBMCs

Blood samples from 37 kidney transplant patients and six age-matched healthy controls (median age 57 years) were analyzed. All volunteers were CMV IgG positive prior to blood sampling. Patient characteristics are detailed in Table 1. Ten patients suffered from CMV reactivation prior to the ELISpot. Their immunosuppressive regimen and age were similar to patients without CMV reactivation. This study was approved by the Institutional Review Board, and all participants provided written informed consent. Peripheral blood mononuclear cells (PBMCs) were separated from 20 ml heparinized blood with Ficoll-Paque (GE Healthcare, Uppsala, Sweden) following the manufacturer's instructions. The isolated PBMCs were suspended in the AIM V medium (Gibco, Grand Island, USA) and counted using the Sysmex hematology analyzer (Sysmex, Kobe, Japan). Due to the limited amount of PBMCs, in the majority of patients, only a subset of the experimental conditions could be tested.

ELISpot assays were performed to measure the IFN-*γ*, IL-21, and IL-17A secretion after treatment with specific antibodies blocking PD-L1, PD-1, and BTLA. ELISpot stripes precoated with the respective antibodies were used (T-Track® ELISpot kit human, Lophius Biosciences, Regensburg, Germany). For the measurement of IFN-*γ* and IL-21, 2 × 10^5^ freshly isolated lymphocytes were stimulated with the CMV-specific antigens IE-1 and pp65, with a CMV lysate and a HEL-299 lysate (all 1 : 25 dilution, Lophius Biosciences) and with phytohemagglutinin (PHA, 1 *μ*g/ml; Remel, Lenexa, USA), which served as positive control. In all experiments, the positive control was valid, defined as more than 150 spots per well. PD-L1 (Thermo Fisher Scientific, San Diego, USA) or BTLA antibodies (BioLegend, San Diego, USA) were added to the cell cultures at concentrations of 0.1, 1, or 10 *μ*g/ml and PD-1 antibodies (Thermo Fisher Scientific, San Diego, USA) at concentrations of 0.5, 1, or 5 *μ*g/ml. Unstimulated cells and cells grown without antibodies served as controls. The cells were incubated for 19 h at 37°C and 5% CO_2_. For the measurement of IL-17A, 4 × 10^5^ cells were stimulated with the same antigens, and the respective antibodies were added. However, cells were preincubated in round bottom plates for 24 h and were thereafter transferred to ELISpot stripes for 48 h. In pilot tests, these conditions could be defined as optimal. After incubation, all stripes were washed and stained using the manufacturer's instructions. The spots were counted by an ELISpot reader (AID, Straßberg, Germany).

### 2.2. Statistical Analysis

Graphic and statistical analyses were performed using GraphPad Prism 5.03 for Windows (La Jolla, CA, USA). For the calculation of significance, either the one-way ANOVA with a posthoc test (Friedman test with Dunn's test to compare all pairs of columns) or the Wilcoxon matched pairs test was used as appropriate.

## 3. Results

**Figure 1:**
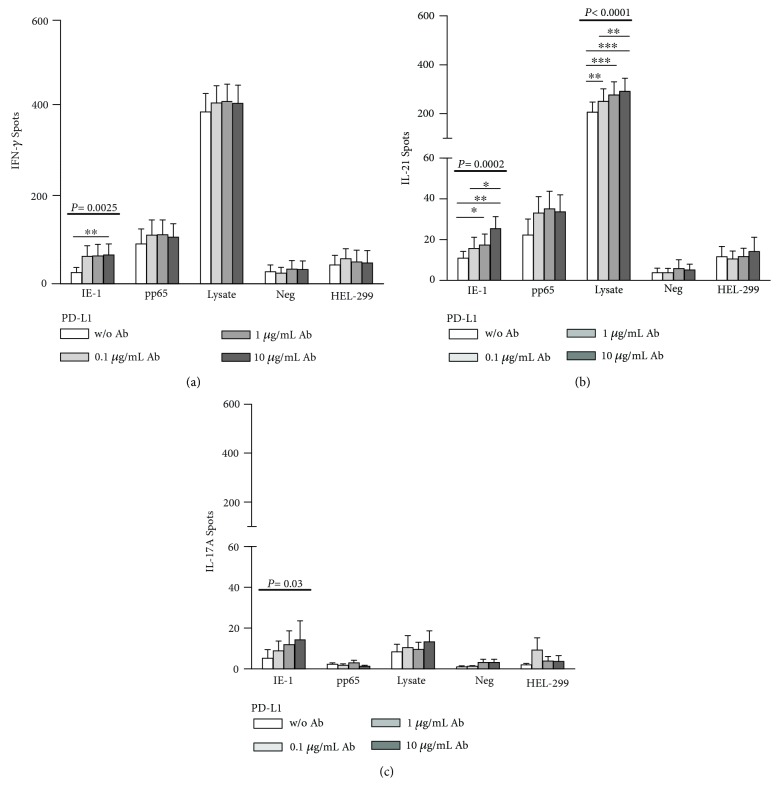
Increase of CMV-specific cytokine secretion by PD-L1 antibodies. The figure shows the IFN-*γ* (a), IL-21 (b), and IL-17A (c) production without and with the addition of PD-L1 antibodies. Lymphocytes of 26 kidney transplant patients, stimulated with CMV IE-1, pp65, CMV lysate (lysate), and HEL-299 lysate, were incubated with the antibody at concentrations of 0.1, 1, and 10 *μ*g/ml. Unstimulated cells (neg) served as a negative control for stimulation with IE-1 and pp65, and the HEL-299 lysate as a negative control for the CMV lysate. IFN-*γ* secretion was determined in 12-18 patients, IL-21 in 13-15 and IL-17A in five patients. Mean and standard of the mean (SEM) of spot numbers are depicted. The effect of PD-L1 antibodies was analyzed by one-way ANOVA with a posthoc test. Values above the bold horizontal lines indicate significant *P* values obtained by the Friedman test, significant results of Dunn's posthoc test (to compare the various concentrations) are given above the thin horizontal lines (^∗^ *P* < 0.05, ^∗∗^ *P* < 0.01, and ^∗∗∗^ *P* < 0.001).

First, a blockade of the ligand PD-L1 was performed to investigate if this results in an enhanced CMV-specific cytokine production. The secretion of IFN-*γ*, IL-21, and IL-17A was measured (Figure 1 and Supplementary file S1).  Figure 1(a) shows an increased IFN-*γ* production after PD-L1 blockade for the cells stimulated with IE-1, pp65, and CMV lysate, which reached statistical significance for IE-1 (*P* = 0.0025). Cells without and with 10 *μ*g/ml of PD-L1 antibody differed significantly (*P* < 0.01).  Figure 1(b) shows that also the IL-21 production is upregulated after treatment with the PD-L1 antibody. This increase was significant for the cells stimulated with IE-1 (*P* = 0.0002) and CMV lysate (*P* < 0.0001). Using IE-1 as stimulus, 1 and 10 *μ*g/ml of PD-L1 antibody led to a significant increase of IL-21 secretion (*P* < 0.05 and *P* < 0.01, respectively). Using the CMV lysate for stimulation, even 0.1 *μ*g/ml of PD-L1 antibody induced a significant increase (*P* < 0.01). For the IL-17A production (Figure 1(c)), an increase of cytokine production was only visible for IE-1-stimulated cells (*P* = 0.03).

**Figure 2:**
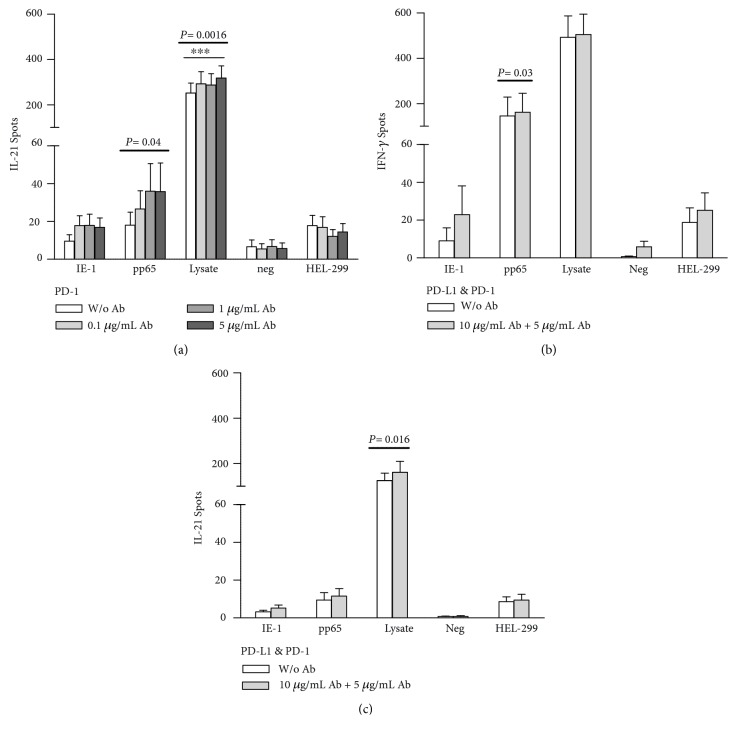
Increase of CMV-specific cytokine secretion by PD-1 and combined PD-L1 and PD-1 antibodies. The figure shows both the IL-21 production without and with the addition of PD-1 antibodies (a) and the IFN-*γ* (b) and IL-21 (c) production without and with the combined addition of PD-L1 and PD-1 antibodies. Lymphocytes of 28 kidney transplant patients were either stimulated with CMV IE-1, pp65, CMV lysate (lysate), or HEL-299 lysate or remained unstimulated (neg). Unstimulated cells (neg) served as a negative control for stimulation with IE-1 and pp65, and the HEL-299 lysate as a negative control for the CMV lysate. For the PD-1 blockade, the antibody was added to the cell cultures at concentrations of 0.5, 1, and 5 *μ*g/ml. For the combined blockade, the PD-L1 antibody was used at a concentration of 10 *μ*g/ml, and the PD-1 antibody at a concentration of 5 *μ*g/ml. IL-21 secretion after addition of PD-1 antibodies was determined in 15 patients, IFN-*γ* and IL-21 production after addition of both antibodies in seven patients each. Mean and standard of the mean (SEM) of spot numbers are depicted. The effect of PD-1 antibodies was analyzed by one-way ANOVA with a posthoc test. Values above the bold horizontal lines indicate significant *P* values obtained by the Friedman test, a significant result of Dunn's posthoc test (to compare the various concentrations) is given above the thin horizontal line (^∗∗∗^ *P* < 0.001). The effect of both antibodies in combination was determined by the Wilcoxon matched pairs test.

In the next experimental series, both a blockade of the receptor PD-1 and a combined blockade of the ligand PD-L1 and the receptor PD-1 were performed (Figure 2 and Supplementary file S2). As PD-L1 had the most prominent effect on IL-21 production, IL-21 was also investigated after the blockade of PD-1 (Figure 2(a)). The results show an increased IL-21 production after the blockade of PD-1, which reached statistical significance for the cells stimulated with pp65 (*P* = 0.04) and CMV lysate (*P* = 0.0016). Using CMV lysate as stimulus, 10 *μ*g/ml of PD-1 antibody led to a significant increase of IL-21 secretion (*P* < 0.001). For the combined blockade of PD-L1 and PD-1, the production of IFN-*γ* (Figure 2(b)) and the IL-21 (Figure 2(c)) was investigated. Significantly increased IFN-*γ* production after the combined blockade could be observed for the cells stimulated with pp65 (*P* = 0.03). Likewise, following stimulation with the CMV lysate, the IL-21 secretion was significantly upregulated after the combined blockade (*P* = 0.016).

In addition, experiments were performed to investigate if a blockade of BTLA also leads to an enhanced CMV-specific cytokine production. The results indicate only a minor increase of IL-21 and IFN-*γ* production after blockade of BTLA (not shown).

**Figure 3:**
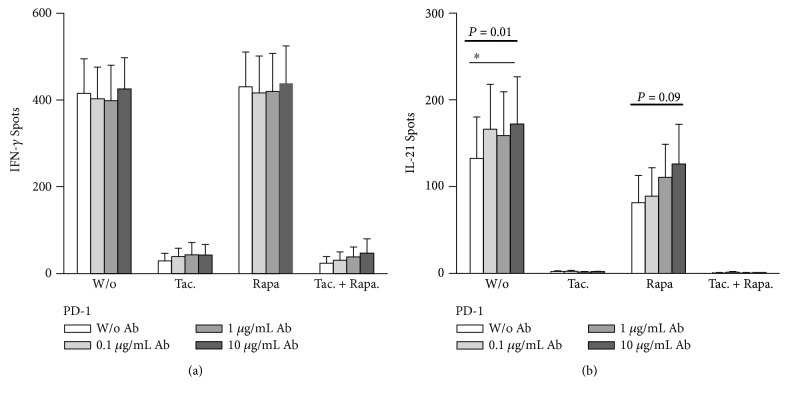
Increase of CMV-specific cytokine secretion by PD-L1 in healthy controls and effect of tacrolimus and rapamycin. The figure shows the production of IFN-*γ* (a) and IL-21 (c) without and with addition of PD-L1 antibodies in healthy CMV-positive controls. Lymphocytes were stimulated with CMV lysate. Stimulation with HEL-299 lysate served as negative control, resulting in average spot numbers of 2 per well (data included in the Supplementary file S3). The cells were grown without immunosuppressive drug or treated with tacrolimus (Tac.), rapamycin (Rapa.), or both drugs in combination (Tac.+Rapa.) and incubated with PD-L1 antibodies at concentrations of 0.1, 1, and 10 *μ*g/ml. IFN-*γ* and IL-21 secretion was determined in six healthy controls. Mean and standard of the mean (SEM) of spot numbers are depicted. The effect of PD-L1 antibodies was analyzed by one-way ANOVA with a posthoc test. Values above the bold horizontal lines indicate *P* values obtained by the Friedman test, a significant result of Dunn's posthoc test (to compare the various concentrations) is given above the thin horizontal line (^∗^ *P* < 0.05).

Finally, the effect of immunosuppressive drugs combined with an inhibition of the PD-L1/PD-1 pathway was studied. The blockade of PD-L1 was repeated with lymphocytes of six CMV-positive healthy controls (Figure 3 and Supplementary file S3). The cells were stimulated with CMV lysate and HEL-299 lysate (negative control) and treated without or with tacrolimus (3 ng/ml), rapamycin (12.5 ng/ml), and tacrolimus and rapamycin in combination. We have chosen to stimulate the cells with the CMV lysate because it induced the strongest response.  Figure 3(a) shows that after PD-L1 blockade the CMV-specific IFN-*γ* production was not changed significantly. However, when treated with tacrolimus we observed a minor, concentration-dependent increase after PD-L1 blockade. The treatment with tacrolimus and the combination of tacrolimus with rapamycin led to a strong downregulation of IFN-*γ* production, whereas rapamycin alone had no effect.  Figure 3(b) shows an increase of CMV-specific IL-21 production after blockade of PD-L1 for two conditions: cells without immunosuppressive agent (*P* = 0.01) and treated with rapamycin (*P* = 0.09). The treatment with tacrolimus and with tacrolimus and rapamycin in combination resulted in a strong decrease of IL-21 production. After treatment with rapamycin alone, IL-21 production was partly preserved. Taken together, effects of PD-L1 on CMV-specific cytokine production could be detected despite the addition of tacrolimus and rapamycin. Treatment with tacrolimus led to an obvious reduction of CMV-specific IFN-*γ* and IL-21 production.

## 4. Discussion

The current study indicates that the modulation of costimulation (especially an inhibition of the PD-1 pathway) leads to an enhancement of CMV-specific cytokine production. Thus, the function of CMV-specific cells in kidney transplant recipients could be partly restored. A blockade of the ligand PD-L1 resulted in an increased IFN-*γ*, IL-21, and IL-17A secretion. The strongest effect could be shown for cells stimulated with IE-1, which is the weakest antigen used. Low level cytokine production could be increased by the blockade of negative costimulation. On the contrary, stimulation with pp65 and CMV lysate caused overall stronger stimulation. Thereby, restoration of cellular immune function by blockade of costimulation may have been partly masked. By blockade of the receptor PD-1, the production of IL-21 could be enhanced distinctly. In addition, the combined blockade of ligand and receptor resulted in an upregulation of the IFN-*γ* and IL-21 production. However, the expected effect of a strong increase, in comparison to the usage of just one antibody, could not be shown. Experiments in healthy controls could confirm the results of the kidney transplant recipients and could further demonstrate that treatment with the immunosuppressive drug tacrolimus resulted in a downregulation of cytokine production.

La Rosa et al. could show that PD-1 expression is upregulated in transplant patients in comparison to healthy individuals, which explains a disturbed CMV-specific immunity in RTX patients [21]. In line with these flow cytometric data, we were able to detect PD-1 expression on unstimulated T-cells of our patient cohort. Furthermore, Sester et al. [9] described that a significantly higher proportion of CMV-specific CD4 T-cells was PD-1 positive in kidney transplant recipients with CMV viremia as compared to nonviremic transplant patients or controls. Our results indicate that CMV-specific cytokine production can be restored by blocking the PD-1 pathway in RTX patients. Accordingly, the enhancement of CMV-specific cytokine production was not that distinct in healthy individuals when no immunosuppressive drugs were added. In comparison, a more distinct upregulation of cytokine production could be shown for healthy individuals when tacrolimus and rapamycin were added to the cell cultures.

These findings correspond to the data of Dirks et al., who demonstrate that a combined blockade of PD-L1 and PD-L2 results in a strong increase of IFN-*γ* production. However, they could not show a difference of cytokine production after isolated blockade of PD-L1 [16]. In contrast to our study, Dirks et al. performed an ELISA to measure cytokine production. As our results exhibit a difference of IFN-*γ* secretion after isolated PD-L1 blockade, it could be assumed that the ELISpot method is more sensitive to demonstrate differences in cytokine production. In addition, our results include the measurement of IL-21 and IL-17A secretion, a blockade of PD-1, and a proof of concentration dependence.

Despite the fact that in kidney transplant recipients a blockade of the PD-L1/PD-1 pathway enhanced CMV-specific Th1, Th21, and Th17 cell responses and that this blockade may help to control CMV reactivation, it currently appears hard to translate these findings into the clinical care of transplant patients. PD-L1 or PD-1 antibodies have already been applied for hematological malignancies [22–24]. Furthermore, SIV-infected monkeys were successfully treated with these antibodies [25, 26]. In addition, Gardiner et al. demonstrated that monoclonal antibodies to PD-1 (BMS-936558) could decrease viral RNA in patients with chronic hepatitis C virus (HCV) infection [27]. However, the induction of alloresponses seems to be a major obstacle impeding clinical application. A study by Boils et al. demonstrated that the usage of the PD-1 pathway inhibitor nivolumab contributed to allograft rejection in a renal transplant patient with stage IV squamous non-small-cell lung cancer [28]. In addition, Kwatra et al. reported allograft rejection in a renal transplant patient with melanoma after treatment with pembrolizumab, a PD-1 inhibitor [29]. Besides allograft rejection, CMV reactivation could be a possible side effect triggered by the usage of checkpoint inhibitors, as described recently by Lankes et al. [30]. They reported that treatment with ipilimumab, a PD-1 inhibitor, led to CMV reactivation in a patient with metastatic melanoma. Likewise, Franklin et al. could show an occurrence of CMV reactivation after treatment with checkpoint inhibitors in patients with metastatic malignant melanoma [31]. Clearly, further experiments are required, but the usage of antibodies with weak affinity to PD-1 could be considered as a therapy option. As these antibodies would only influence cells with high PD-1 expression, they could act more selectively, which could reduce the risk of allograft rejection.

## 5. Conclusions

Our study could show for the first time that the blockade of the PD-L1/PD-1 pathway also modulates CMV-specific Th21 and Th17 cell function in kidney transplant recipients. It is now mandatory to clarify the role of Th21 and Th17 cells in CMV control of these patients.

## Figures and Tables

**Table 1:** Patient demographics.

Total (no.)	37
Sex (no.)	
Male	22
Female	15
Median age (range) (yr)	56 (19-80)
CMV serostatus prior to TX (no.)	
D-/R-	1
D-/R+	11
D+/R+	22
D+/R-	3
First/repeated transplantation (no.)	32/5
Deceased/living donor	27/10
Severe infection post TX^a^	17
Acute rejection	15
Chronic rejection	15
Graft failure	6
Immunosuppressive regimen (no.)^b^	
CNI, MMF, and steroids	25
CNI, mTOR, and steroids	3
CNI and mTOR	2
CNI, AZA, and steroids	2
CNI and steroids	3
Other	2
Median interval TX-ELISpot (range) (months)	28 (1-520)
Median number of CMV reactivations^c^ (range)	0 (0-4)
With/without treatment with valcyte^b^ (no.)	4/33

AZA: azathioprine; CNI: calcineurin inhibitor; MMF: mycophenolate mofetil; mTOR: mammalian target of rapamycin; TX: transplantation. Of note, none of the kidney transplants was placed on the pump prior to transplantation. ^a^Severe infections such as sepsis, pneumonia, or meningitis. ^b^At the time of the ELISpot assay. ^c^Episodes with viral load > 500 copies/ml prior to the ELISpot assay.

## Data Availability

The Excel data used to support the findings of this study are included within the supplementary information file.

## Supplementary Materials

Supplementary Materials The raw data corresponding to Figures 1, 2, and 3 of this study are included within the Supplementary files S1-S3.
